# A proposed stratification system to address the heterogeneity of Subdural Hematoma Outcome reporting in the literature

**DOI:** 10.1007/s10143-024-02444-7

**Published:** 2024-05-07

**Authors:** Peyton L. Nisson, John Francis, Michelot Michel, Takuma Maeda, Chirag Patil

**Affiliations:** 1 Department of Neurosurgery, Cedars-Sinai, 127 S. San Vicente Blvd., Ste. A6213, Los Angeles, CA USA; 2 School of Medicine, Case Western Reserve University, Cleveland, OH USA; 3 College of Medicine, University of Florida, Gainesville, FL USA; 4 Department of Translational Neuroscience, Barrow Neurological Institute, Phoenix, AZ USA

**Keywords:** Subdural hematoma, Subdural hematoma evacuation, Stratification system, Traumatic brain injury, Outcomes

## Abstract

A major challenge within the academic literature on SDHs has been inconsistent outcomes reported across studies. Historically, patients have been categorized by the blood-product age identified on imaging (i.e., acute, subacute, or chronic). However, this schematic has likely played a central role in producing the heterogeneity encountered in the literature. In this investigation, a total of 494 patients that underwent SDH evacuation at a tertiary medical center between November 2013-December 2021 were retrospectively identified. Mechanism of injury was reviewed by the authors and categorized as either positive or negative for a high-velocity impact (HVI) injury. Any head strike injury leading to the formation of a SDH while traveling at a velocity beyond that of normal locomotion or daily activities was categorized as an HVI. Patients were subsequently stratified by those with an acute SDHs after a high-velocity impact (aSDHHVI), those with an acute SDH without a high-velocity impact injury (aSDHWO), and those with any combination of subacute or chronic blood products (mixed-SDH [mSDH]). Nine percent (n = 44) of patients experienced an aSDHHVI, 23% (n = 113) aSDHWO, and 68% (n = 337) mSDH. Between these groups, highly distinct patient populations were identified using several metrics for comparison. Most notably, aSDHHVI had a significantly worse neurological status at discharge (50% vs. 23% aSDHWO vs. 8% mSDH; p < 0.001) and mortality (25% vs. 8% aSDHWO vs. 4% mSDH; p < 0.001). Controlling for gender, midline shift (mm), and anticoagulation use in the acute SDH population, multivariable logistic regression revealed a 6.85x odds ratio (p < 0.001) for poor outcomes in those with a positive history for a high-velocity impact injury. As such, the distribution of patients that suffer an HVI related acute SDH versus those that do not can significantly affect the outcomes reported. Adoption of this stratification system will help address the heterogeneity of SDH reporting in the literature while still closely aligning with conventional reporting.

**Supplementary Information:**

The online version contains supplementary material available at 10.1007/s10143-024-02444-7.

## Introduction

**Fig. 1:**
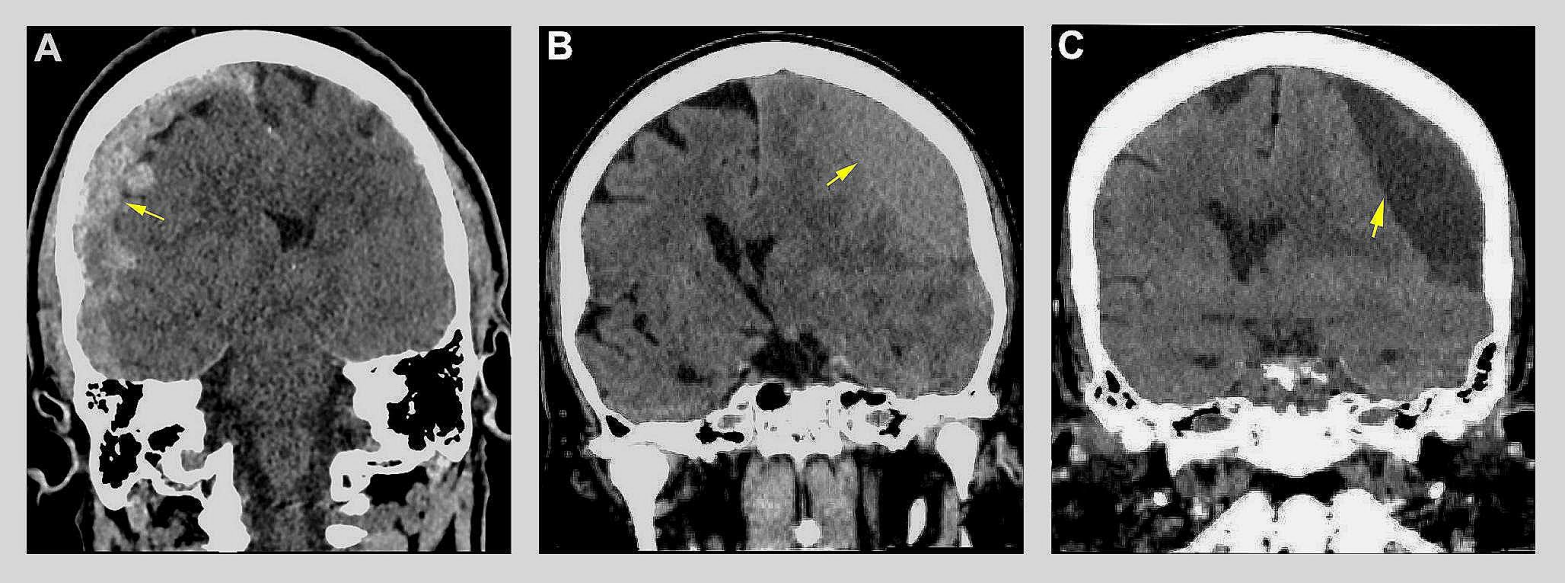
Conventional categorization of subdural hematomas. In this panel of computed tomography coronal images, the three types of subdural hematomas are illustrated using conventional reporting. Figure **1A** shows an acute subdural hematoma with a hyperdense appearance relative to brain tissue while a subacute subdural hematoma is isodense (Fig. **1B**). A chronic subdural hematoma is hypodense relative brain tissue and similar in appearance to cerebrospinal fluid (Fig. **1C**)

Subdural hematomas (SDH) represent one of the most common intracranial mass-lesions associated with high morbidity and mortality [1]. Predominately a disease of the elderly, recent projections indicate 1.5 billion individuals will be over 65 by 2050, with over 80 representing the fastest growing segment of the population in developed countries [2]. Historically, SDHs have been categorized by the blood product age, i.e., acute, subacute, and chronic (Fig. 1) [3, 4]. However, the differences that exist between SDH types are not limited to exclusively their radiographic densities, which may have only a small role in the complex pathophysiology pathway from which they develop. The majority of SDHs occur secondary to traumatic brain injury (TBI) [5]. Traumatic brain injuries can vary greatly in both their mechanism of injury and severity. Herein arises the challenge in categorizing these varied patients. Despite sharing blood products identified on computed tomography (CT), conventional reporting does not allot any measure towards the magnitude of TBI. Traumatic brain injuries unto themselves are associated with numerous physiologic changes and downstream sequalae with the potential to have devastating neurological outcomes. Although a SDH is often easily recognized on imaging, it still does not adequately represent the underlying injury which may be occurring on a molecular or cellular level [6]. Consequently, an assortment of intracranial pathology is frequently categorized together under a single designation.

A major challenge within the academic literature on SDHs has been inconsistent outcomes reported across studies, most notably for older age groups with acute hemorrhages [7]. Mortality rates have ranged from 40 to 90% for acute SDHs in select populations though more recent reports since 2010 have ranged from 15 to 67.2% for elderly patients [8–12]. Admittedly, there will always be some variation between studies, but such extreme differences have led to contradictory recommendations in the literature and underscore a greater issue at hand [10, 13–15]. In this investigation, we sought to elucidate upon the unique subpopulations being combined in the current conventional reporting of SDHs and proposed a stratification system to help standardize and improve reproducibility of future research on this topic.

## Materials & methods

At a single, tertiary academic medical center, patients who underwent subdural hematoma evacuation between November 2013 and December 2021 were retrospectively identified using ICD 9 and 10 billing codes. Patients of any age with a SDH requiring surgical evacuation were included. In the event a single patient had multiple hospital admissions, only their first encounter was included. Initially 689 patients were captured, 195 of whom were excluded because of duplicate admissions, epidural hematoma evacuation, or no surgery transpired. In this retrospective, cohort study, data was retrieved from the electronic medical record included basic demographics, pertinent past medical history, method of arrival to the hospital, mechanism of injury, Glasgow coma scale (GCS) at time of arrival and discharge, length of stay (LOS), subdural hematoma size, laterality, presence of midline shift, surgery type, inpatient mortality, and neurological outcome at the time of discharge using the Glasgow Outcome Scale (GOS). Any GOS scores 1–3 were categorized as a “poor outcome.” In the ad hoc analysis, patients with a GCS score 8 or less at the time of arrival were categorized as having a “poor GCS score.” Subdural hematomas were classified as acute if the fluid collection was primarily hyperdense relative to the brain parenchyma and chronic if hypodense. Those with a mixed density or isodense were categorized as subacute. Surgery types were categorized as craniotomy, burr hole craniostomies, and craniectomy.

Mechanism of injury was reviewed by the authors and categorized as either positive or negative for a high-velocity impact (HVI) injury. Any head strike injury leading to the formation of a SDH that was incurred while traveling at a velocity beyond that of normal locomotion or daily activities was categorized as HVI. A fall from 10 feet or higher or motor-vehicle versus pedestrian was also counted as positive. Conversely, any assaults or a fall down a set of stairs were not. A list of presentations categorized as HVI are listed in Table 1. Using the proposed stratification system (Table 2), patients with an acute SDH were categorized by those with a HVI (aSDH_HVI_) in their history and those without (aSDH_wo_). Any patients with a subacute or chronic subdural hematoma were categorized as a mixed-SDH (mSDH).

**Table 1:** List of presentation types categorized as high velocity impacts injuries

High-Velocity Impact Injury	Number
Motor Vehicle Versus Pedestrian	28
Motor Vehicle Collision	21
Fall from over 10 feet	4
Motorcycle Collision	3
Motor Vehicle versus Bicycle	3
Bicycle Crash	2
MVC Roll Over	2
Dirt Bike Accident	1
Skiing Accident	1
Scooter versus Pole	1
Total	66

**Table 2:** Proposed stratification system for subdural hematomas. Abbreviations: aSDH_HVI_; acute subdural hematoma with high-velocity impact, aSDH_wo_; acute subdural hematoma without high-velocity impact

Proposed Classification	aSDH_HVI_	aSDH_wo_	mSDH
Acute SDH			
History w/ High Velocity Mechanism of Injury	**+**		
History w/o High Velocity Mechanism of Injury		**+**	
Subacute Blood Products			**+**
Chronic Blood Products			**+**

### Statistical analysis

Patient groups were compared using Student’s t-test for continuous parametric data. *X*^2^ was used for comparing categorical variables; if the expected frequency for an observation was less than 5 the Fisher exact test was used. One-way ANOVA test was used to compare the more than 2 continuous variables. Univariate logistic regression was used to compare the odds ratio of each proposed strata for the dependent variables “poor outcome” and “mortality”. A backwards stepwise multivariable logistic regression analysis was conducted in the acute SDH population with inclusion criteria set at *p*-value of less than 0.10 for the model. In an ad hoc analysis, the prognostic value of poor GCS score at time of arrival was compared to aSDH_HVI_ by substituting aSDH_HVI_ with poor GCS in the same multivariable logistic regression model. Since these two variables were not mutually independent, they could not be included in the same regression model without violating the statistical assumption for an absence of multicollinearity. The odds ratio for each respective variable and the model’s overall predictive accuracy using the area under the ROC curve were then compared. Only a *p*-value equal or less than 0.05 was considered significant and any missing observations were left blank during analysis. Statistical analysis was performed using STATA 14 (StataCorp LP, College Station, Texas). Approval from the hospital’s institutional review board to perform this clinical study and waiver of patient consent for data collection was obtained.

## Results

A total of 494 patients with SDHs requiring evacuation were included in the study. The mean age was 68 years (Range 19 to 105) with a male predominance of 72%. The mean SDH size was 19 mm (Range 5–40 mm); midline shift was present in 81% of patients. The majority of SDHs were chronic (46%), followed by acute (32%), and subacute (22%). A total of 87 (18%) patients were on anticoagulation at the time of presentation. A detailed comparison of patient demographics, subdural hematoma characteristics, and co-morbidities by conventional SDH reporting is provided in Supplemental Table 1.

### High-velocity impact and subdural hematomas

A total of 66 (13%) patients with clinical histories positive for HVI. Acute SDHs were stratified between those positive for a HVI history (aSDH_HVI_) to those without (aSDH_wo_) and those with mSDHs as shown in Table 3. aSDH_HVI_ most often presented by ambulance (96% vs. 45% aSDH_WO_ vs. 19% mSDH; *p* < 0.001), poor GCS 3–8 at arrival (52% vs. 21% aSDH_WO_ vs. 1% mSDH; *p* < 0.001), longer LOS (31 days vs. 18 days aSDH_WO_ vs. 10 days mSDH; *p* < 0.001), Trach/PEG placement (40% vs. 13% aSDH_WO_ vs. 5% mSDH; *p* < 0.001), worse neurological status at discharge (43% vs. 25% aSDH_WO_ vs. 8% mSDH; *p* < 0.001) and greater mortality (25% vs. 8% aSDH_WO_ vs. 4% mSDH; *p* < 0.001). Patients with aSDH_HVI_ exhibited 2.32x OR (95% CI 1.14–4.7, *p* = 0.02) for poor outcomes and 2.9x OR (95% CI 1.09–7.75, *p* = 0.03) for death compared to aSDH_wo_.

**Table 3:** Pertinent patient presentation and outcome factors by subdural hematoma type using the proposed stratification system. *Poor Outcomes were defined by a Glasgow Outcome Scale 1 to 3 at the time of discharge. Abbreviations: GCS; Glasgow Coma Scale, OSH; outside hospital transfer; Trach/PEG; tracheostomy/percutaneous endoscopic gastrostomy; SD; standard deviation, aSDH_wo_; acute subdural hematoma without high-velocity impact; aSDH_HVI_; acute subdural hematoma with high-velocity impact

	aSDH_HVI_	aSDH_wo_	*p*-value	aSDH_wo_	Mixed	*p*-value	Subacute	Chronic	*p*-value
Number	44	113		113	337		110	227	
Symptom Duration in days (SD)	1.3 (2)	2.2 (4)	0.10	2.2 (4)	9.5 (17)	**< 0.001**	10.2 (16)	9.2 (17)	0.6
Mechanism of Arrival									
Ambulance	42 (96)	51 (45)	**< 0.001**	51 (45)	65 (19)	**< 0.001**	16 (15)	49 (22)	
Personal Vehicle	2 (4)	25 (22)		25 (22)	158 (47)		57 (52)	101 (45)	0.43
OSH transfer	0	5 (4)		5 (4)	90 (27)		29 (26)	61 (27)	
Unknown	0	32 (28)		32 (28)	24 (7)		8 (7)	16 (7)	
GCS on Arrival									
3 to 8	23 (52)	24 (21)	**< 0.001**	24 (21)	2 (1)	**< 0.001**	1 (1)	1 (0)	
9 to 12	4 (9)	14 (12)		14 (12)	19 (6)		7 (6)	10 (4)	0.43
>12	17 (3)	75 (66)		75 (66)	316 (94)		102 (93)	214 (94)	
GCS at Discharge									
3 to 8	16 (36)	10 (9)	**0.001**	10 (9)	11 (3)	**0.005**	3 (3)	8 (4)	
9 to 12	2 (5)	5 (4)		5 (4)	4 (1)		1 (1)	3 (1)	1
>12	26 (59)	98 (87)		98 (87)	322 (96)		106 (96)	216 (95)	
Length of Stay days (SD)	31 (34)	18 (21)	**0.004**	18 (21)	10 (13)	**< 0.001**	8.5 (8.5)	10.8 (15)	0.14
Trach/PEG Placement	21 (40)	13 (13)	**< 0.001**	13 (13)	16 (5)	**0.001**	4 (4)	12 (5)	0.6
Reoperation	1 (2)	11 (10)	0.18	11 (10)	42 (13)	0.44	18 (16)	24 (11)	0.14
Inpatient Mortality	11 (25)	9 (8)	**0.004**	9 (8)	12 (4)	0.06	3 (3)	9 (4)	0.76
Poor Outcome	22 (50)	26 (23)	**0.001**	26 (23)	27 (8)	**< 0.001**	8 (7)	19 (8)	0.75

**Fig. 2:**
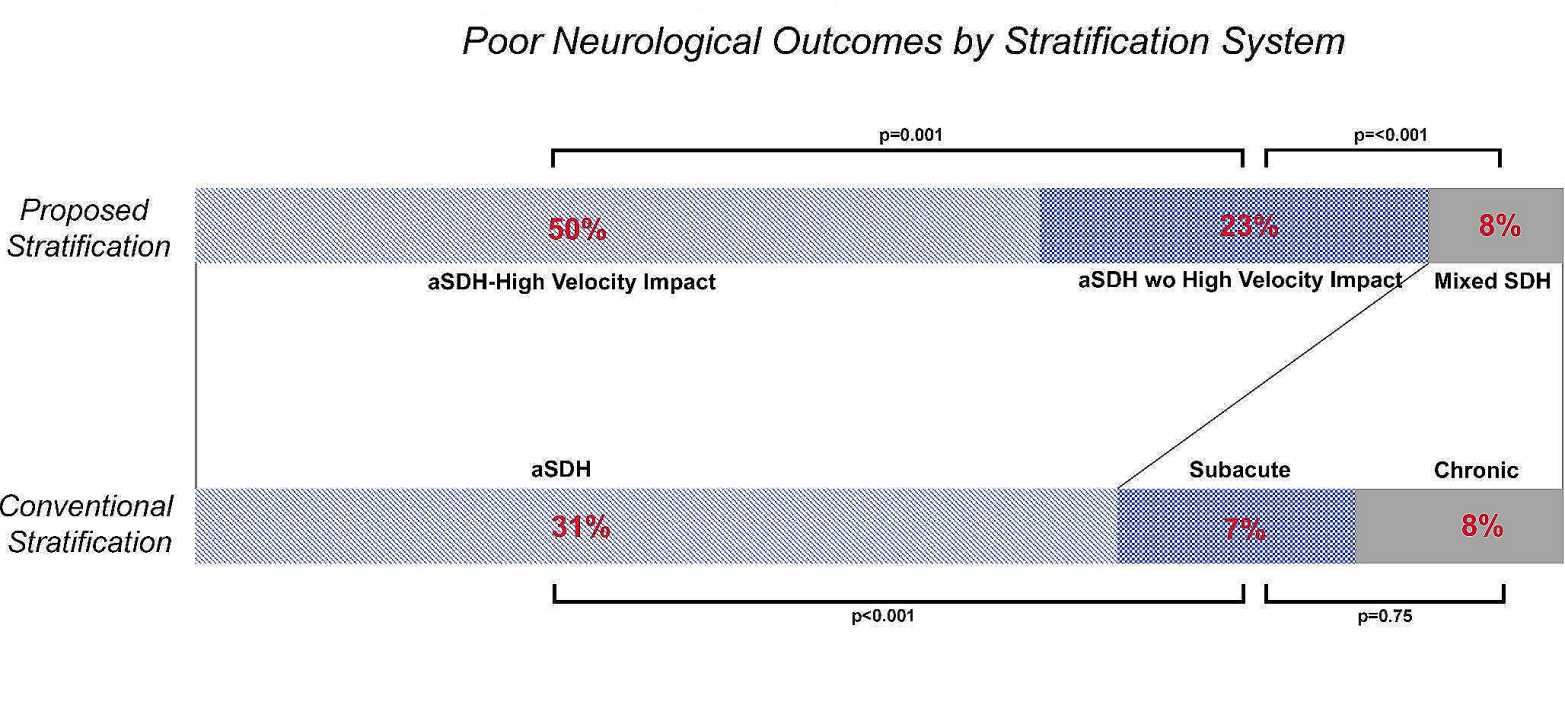
A distribution of poor neurological outcomes by the two different stratification systems is listed. Poor outcomes were categorized as Glasgow Outcome Scale 1–3. The upper bar represents the proposed stratification system. The light blue represent acute subdural hematoma patients with a high-velocity impact mechanism of injury (aSDH_HVI_). The dark blue block is acute subdural hematomas without HVI (aSDH_wo_). The beige colored block represents mixed-SDH (mSDH). The lower bar is the conventional reporting system with the light blue block representing acute subdural hematoma. The dark blue block represents subacute subdural hematoma. The beige block represents chronic subdural hematoma. A significantly greater rate of poor outcomes (*p* = 0.001) for patients with aSDH_HVI_ than aSDH_wo_ which would ordinarily be reported as a single group using the conventional system. Despite having these patients separated from the acute SDH population, the aSDH_wo_ group still exhibited a significant difference in outcomes from those with mSDHs (*p* < 0.001) while none was found between subacute and chronic SDHs (*p* = 0.75)

**Fig. 3:**
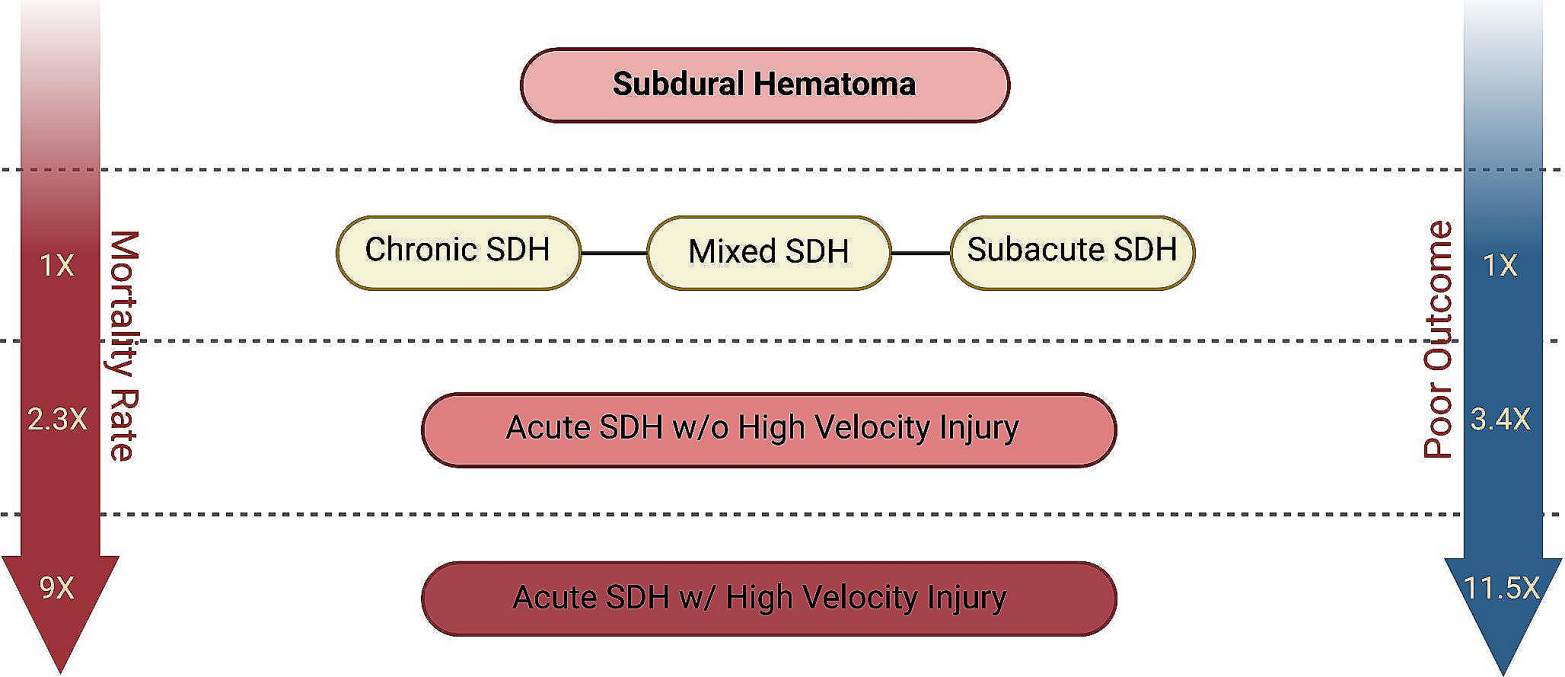
Figure 3 depicts the results of the univariate logistic regression analysis for poor outcomes and mortality between each proposed SDH strata. The arrows represent the increasing odds ratio for each outcome using mSDH as the standard value. The red arrow on the left, which represents mortality rate, shows a 2.3 OR (95% CI 0.96–5.71, *p* = 0.06) for aSDHwo and 9.0 OR (95% CI 3.70-22.05, *p* < 0.001) for aSDH_HVI_ patients. For poor outcome, aSDH_wo_ had 3.4 OR (95% CI 1.9–6.2, *p* < 0.001) and aSHW_HVI_ 11.5 (95% CI 5.64–23.3, *p* < 0.001). Abbreviations: SDH; subdural hematoma, w/o; without, w; with

High-velocity impact had no association with poor outcomes (0% HVI group vs. 9% in w/o HVI group; *p* = 0.24) or death (0% HVI vs. 4% w/o HVI; *p* = 1) in patients with non-acute hemorrhages. Similarly, no difference in the symptom duration, mechanism of arrival, GCS scores on arrival or at discharge, or any other of the metrics were found between subacute and chronic SDH groups. A comparative distribution of poor outcomes between each group in the proposed stratification system is provided in Fig. 2. Next, the odds ratio for poor outcome and mortality was calculated for each strata using univariate logistic regression. The mSDH group was set as the baseline value for comparison. The results are listed in Fig. 3. All were significant with a *p*-value < 0.001 except for the mortality rate of aSDH_wo_ compared to mSDH (OR 2.3, *p* = 0.061).

### Multivariable regression analysis in the acute SDH population

A backwards stepwise multivariable logistic regression analysis in the acute SDH population was performed with poor neurological status at the time of discharge as the dependent variable. Age at the time of surgery, gender, SDH size (mm), midline shift (mm), and HVI were initially evaluated. Only gender, midline shift (mm), anticoagulant use, and HVI met the inclusion criteria for the model. Female patients were less likely to experience poor outcomes than males (OR 0.34, 95% CI 0.15–0.75, *p* = 0.007) while midline shift (mm) (OR 1.08, 95% CI 1.00-1.17, *p* = 0.043), anticoagulant use (OR 3.24, CI 1.2–8.7, *p* = 0.019) and HVI (OR 6.85, CI 2.7–17.2, *p* < 0.001) were risk factors for poor outcomes. The AUROC demonstrated good predictive accuracy with a value of 0.74 and the Hosmer-Lemeshow goodness-of-fit test indicated a good fit (*p*-value 0.28).

### Ad hoc analysis: glasgow coma scale versus high-velocity impact

An ad hoc analysis that compared outcomes using a GCS scoring system and the proposed HVI stratification system was conducted. Patients were categorized as having either a poor GCS score (***≤*** 8) or favorable GCS (> 8) in the aSDH population. A total of 47 patients comprised the poor GCS group while 110 were in the favorable group. There were 23 patients with cross-over between the poor GCS and aSDH_HVI_ groups as they were not mutually exclusive. The rates of the aSDH_HVI_ group versus the poor GCS group for each outcome metric are listed, respectively: arrival by ambulance (96% vs. 79%), mean length of stay (31 days vs. 27 days), trach/PEG placement (42% vs. 37%), poor outcomes (50% vs. 47%), reoperation rate (2% vs. 2%), and inpatient mortality (25% vs. 19%). Using the same multivariable logistic model as above (gender, midline shift (mm), and anticoagulation), HVI was substituted with poor GCS. Poor GCS had a odds ratio of 3.70 (95% CI 1.62–8.45, *p* = 0.002). The AUROC was 0.71 and Hosmer-Lemeshow goodness-of-fit test *p*-value of 0.89.

## Discussion

As demonstrated in this study, SDH patients represent a highly heterogenous group. Despite possessing a range of presentation types and varying degrees of neurologic injury, SDH outcomes are often reported almost exclusively by the age of the blood products at the time of presentation. Although this method has its merits, this nomenclature has had unintended, adverse effects in the literature. Insufficient stratification of these subpopulation groups has contributed to conflicting conclusions across studies, making it is difficult to develop a consensus of opinion or treatment guidelines. This is particularly relevant for the advanced geriatric population with acute SDHs where a call for guidelines regarding the role for surgery and possible rationing of care has been made [9, 13]. With an anticipated surge in both the age and number of patients with SDHs requiring evacuation, the need for more accurate and reliable outcome reporting becomes greater each year [16].

Through incorporating a schematic that stratified groups by combining HVI and acute blood products, we were able to delineate upon two distinct patient populations that would have traditionally been shrouded under a single, acute SDH designation. For instance, those with aSDH_HVI_ arrived at the hospital more often by ambulance (96% vs. 45%, *p* < 0.001), had a significantly longer hospital stay (31 vs. 18 days, *p* = 0.004), over double the rate of poor outcomes (50% vs. 23%, *p* = 0.001), and over triple the mortality (25% vs. 8%, *p* = 0.004) compared to the aSDH_wo_ group. Outcomes of the aSDH_wo_ also remained distinct from with mSDH group, demonstrating a difference in each metric listed above as well (Fig. 3). Among the acute SDH population, HVI alone carried over a 500% increased risk for poor outcomes (OR 6.85, *p* < 0.001) controlling for patient gender, midline shift, and anticoagulation use. The association of female gender, midline shift, and anticoagulation use corroborate prior studies that also have reported these as risk factors for poor outcomes [17–19].

### Heterogeneity of Subdural Hematoma Outcome reporting

Until now, no prior studies have proposed a method to address the heterogeneity of SDH outcomes reported in the literature, to the best of our knowledge. A minority have attempted to stratify SDH outcomes based on traumatic injury type, presenting neurological condition, or GCS score [20–23]. While some SDH scoring systems exist, they have been aimed primarily at prognosticating and guiding treatment decision-making [24–26]. Consequently, there exists a need for a more specific and standardized categorization technique for SDHs beyond that of blood-age chronicity [27].

Surgical intervention of SDH has become controversial due to concerns for efficacy in the face of alarmingly high poor outcomes and mortality in the advanced geriatric population [13, 28]. Benedetto et al. (2017) reported a 6-month mortality rate of 67.2% in a series of 67 patients over 70 years with acute SDHs surgically evacuated [9]. Petridis et al. (2009) reported an inpatient mortality of 53.8% in a series of patients 65 or older [29]. For chronic SDHs, Whitehouse et al. (2016) reported 15 times the rate of poor outcomes of mortality for inpatient death for those 75 or older [30]. However, other studies reporting nearly half or less the rate of poor outcomes also exist around the same time period [15]. Won et al. (2017) found an inpatient mortality of only 28% in patients over 80 with aSDHs [10]. Younsi et al. (2021) reported an inpatient mortality of 33% for aSDHs [14]. Younger age groups have not been immune to these wide variations either. Lavrador et al. (2018) reported poor functional outcomes in 58% (GOS 1–3, 40/69) of those with aSDH while a series by Ryan et al. (2012) found 88% (184/210) of patients had good outcomes with a GCS score 13–15 at the time of discharge in the aSDH evacuated group [12, 31].

### Causes of Subdural Hematoma Outcome Heterogeneity

Though it is difficult to say with absolute certainty, there are likely numerous factors contributing to these differences. A medical center’s surrounding population, location within a city, and the standards of practice can all be contributors. Relatively small sample sizes on this topic has also been cited as a potential cause [12]. In a large systematic review, Manivanne et al. (2021) cited differences in functional outcome metrics (i.e., GOS, GCS, Markslwalder scale, mRS) and follow-up time as possible contributors for heterogeneity of the pooled data from the literature [13].

An important factor at the crux of these differences is the proportion of patients with severe TBI and those with more isolated SDHs in the acute SDH population. As shown here, the proportion of patients with HVI aSDHs compared to those without has the potential to significantly affect outcomes. Albeit less often, SDHs can occur absent of a TBI event. Rapid acceleration-deacceleration movements with or without head strike can potentially still lead to shearing of bridging veins. Spontaneous SDHs can occur in patients on anticoagulation as well those with intracranial hypotension [32–35].

Among those who experience a TBI of extreme severity, the SDH is often only one of many sources of neurological injury. These may include diffuse axonal injury (DAI), brain contusions, cerebral edema, blood brain barrier disruption, dysautoregulation of cerebral blood flow, changes in cellular metabolism, and surge of neurotransmitter release at neurotoxic levels [36–40]. Prior efforts to stratify patients by neurological exam at the time of presentation or TBI severity but this is not well-standardized or commonly performed [22, 41].

### Devising an effective stratification system

In devising a stratification system that incorporated this information, we found designating those with a HVI injury in the acute SDH population (Table 1) effective. It provided sufficient stratification between patient populations to realize differences spanning several aspects of patients’ hospital course. Though HVI’s also occurred in those with subacute and chronic SDHs, the presence of older blood products in it of itself selected for patients with less severe or without TBIs. This is supported by HVI demonstrating no statistical association with patient functional outcomes (*p* = 0.24) or mortality (*p* = 1) in the mSDH group. Additionally, no differences in presentation types, GCS on arrival, or other outcome metrics in those with subacute and chronic SDHs were found. With the exception of those in the chronic group having greater mean age (74 vs. 65 years, *p* = 0.01), these groups were very similar including the use of burr hole craniostomies versus craniotomies (20% chronic vs. 14% subacute, *p* = 0.12). Consequently, the merging of these blood ages as a single category avoided redundancy as it pertained to general disease course for those being surgically treated. This coincides and supports articles in the literature that have categorized both subacute and chronic SDHs together as “chronic” [27, 42, 43].

Another important feature in this design was keeping the stratification categories simple, with minimal group numbers. Defining a high-velocity mechanism of injury is still relatively intuitive to assign and easily retrievable in the history-of-present-illness. In maintaining a 3-tiered system that keeps blood age as a distinguishing feature, its adaptation is also a relatively minor transition from convention. Granted, there are likely other valuable categorizing features such as concussion grade, specific trauma event, or presenting GCS score. Yet these come at the expense of adding more categories and potentially requiring the retrieval of additional data points. Additional categories stand to lower the denominator across studies and could exacerbate the effect of exaggerated outcomes due to smaller sample sizes [44]. Lastly, concomitant injuries are often sustained with motor-vehicle accidents, pedestrian versus motor vehicle, and other high kinetic energy mechanism of trauma [45, 46]. By stratifying by HVI in the aSDH patient population, this likely better selects for those who sustained these other injuries rather than isolated GCS scores, which can also have a significant effect on outcomes [47].

In the ad hoc analysis, aSDH_HVI_ demonstrated a greater odds ratio (OR 6.8 vs. 3.7) and predictive value for poor outcomes at discharge (AUROC 0.74 vs. 0.71) than poor GCS upon arrival. However, comparing these variables is challenging since they are not independent from one another, and interpretation may be limited. More commonly used statistical methods such as Chi2 or McNamer test cannot be used due to a failure to meet the required assumption for mutual independence [48, 49]. Although demonstrating a correlation with poor outcomes is not the salient metric for evaluating the utility of a stratification system (as listed for the reasons above), these findings do suggest aSDH_HVI_ may provide a better representation of the underlying pathological changes seen in patients with different types of aSDHs. For instance, seizures, post-concussive symptoms, and SDH mass effect may have caused patients to present with a poor GCS score but still experience significant recovery following evacuation. Conversely, irreversible damage associated with DAI, brain contusions, and other sources of neuron injury that may have occurred after a HVI injury could have hindered as rapid or robust of a recovery.

### Limitations

Study limitations include the retrospective study design. The generalizability of our institutional findings may be biased by the hospital location and referral pattern. Future investigation to further validate the utility of this proposed stratification system is needed. It would have been informative to compare the rates of DAI between the HVI and non-HVI patients with aSDHs, but this was not possible as MRIs were routinely performed in this patient population. Additionally, only surgical patients were included in this analysis. It is unclear the utility of this stratification system for non-surgical candidates and warrants further investigation.

## Conclusion

Studies investigating subdural hematomas have historically reported outcomes by grouping patients based on the age of the blood products seen on imaging. Among the innumerable types of presentations that precede a SDH, this approach has insufficiently stratified the varied disease course in patients reported. In turn, this has likely been a major contributing factor in the heterogeneity of patient outcomes reported in the literature. A growing demand for more reliable reporting is needed in light of projected population trends and related public health implications. By categorizing those with aSDH who suffered a HVI to those who did not, and combining those with subacute and chronic SDHs, meaningful distinctions between patient populations based on presentations, hospital course, and outcomes were identified. The HVI designation alone was associated with a greater than 500% risk for poor outcomes at discharge in the acute SDH population. Compared to an alternative stratification method such as poor GCS (***≤*** 8) at the time of arrival, aSDH_HVI_ revealed a greater odds ratio and predictive accuracy for poor outcomes using separate multivariable logistic regression models. This three-tiered system is easily adaptable from current convention and serves as an important step towards addressing the need for more improved consistency for outcome reporting on this topic.

## Electronic supplementary material

Below is the link to the electronic supplementary material.

Supplementary Material 1

## Data Availability

No datasets were generated or analysed during the current study.
