# Supplementary material for: A proposed stratification system to address the heterogeneity of Subdural Hematoma Outcome reporting in the literature
Source: Neurosurg Rev. 2024 May 7;47(1):207. doi: 10.1007/s10143-024-02444-7 (PMC11076356; doi:10.1007/s10143-024-02444-7)
Supplement: Supplementary file 1 — Supplementary Material 1 [file 10143_2024_2444_MOESM1_ESM.docx]

**Supplemental Table 1.** Patient demographic and medical history summary by hematoma blood-product age. Abbreviations: HTN; hypertension, COPD; chronic obstructive pulmonary disease, DM; diabetes mellitus, CAD; coronary artery disease; CVA; cerebrovascular accident; CHF; congestive heart failure, PVD; peripheral vascular disease.

| **Characteristics** | **Acute** | **Chronic** | **Subacute** | **p-value** | **Total** |
| --- | --- | --- | --- | --- | --- |
| **Number of patients** | 157 (32) | 227 (46) | 110 (22) |  | 494 |
| **Mean Age (years)** | 63 | 76 | 72 | **<0.01** | 68 |
| **Age Range (years)** | 19-105 | 22-100 | 28-91 |  | 19-105 |
| **Males** | 100 (64) | 171 (75) | 84 (73) | **0.02** | 355 (72) |
| **Mean SDH size mm (SD)** | 16 (8) | 21 (7) | 20 (8) | **<0.01** | 19 (8) |
| **Laterality** |  |  |  |  |  |
| **Right** | 72 (46) | 74 (33) | 48 (44) | **0.003** | 194 (39) |
| **Left** | 64 (41) | 88 (39) | 45 (41) |  | 197 (40) |
| **Bilateral** | 21 (13) | 63 (18) | 17 (16) |  | 101 (21) |
| **Midline Shift Present** | 133 (86) | 174 (77) | 90 (83) | 0.10 | 397 (81) |
| **Surgery Type** |  |  |  |  |  |
| **Craniotomy** | 113 (71) | 179 (79) | 99 (90) | **<0.01** | 391 (79) |
| **Burr Hole** | 5 (3) | 46 (20) | 38 (14) |  | 61 (12) |
| **Craniectomy** | 39 (25) | 2 (1) | 1 (1) |  | 42 (9) |
| **Medical Comorbidities** |  |  |  |  |  |
| **HTN** | 47 (30) | 96 (42) | 38 (35) | **0.04** | 181 (37) |
| **COPD** | 1 (1) | 6 (3) | 1 (2) | 0.25 | 8 (2) |
| **DM** | 18 (11) | 31 (14) | 17 (16) | 0.62 | 66 (13) |
| **CAD** | 14 (9) | 31 (14) | 9 (8) | 0.19 | 54 (11) |
| **CVA** | 9 (6) | 19 (8) | 4 (4) | 0.23 | 32 (7) |
| **Dementia** | 7 (5) | 7 (3) | 2 (2) | 0.49 | 16 (3) |
| **CHF** | 4 (3) | 11 (5) | 5 (5) | 0.5 | 20 (4) |
| **PVD** | 4 (3) | 6 (3) | 1 (1) | 0.57 | 11 (2) |
| **Aspirin Use (Y)** | 32 (20) | 74 (33) | 28 (26) | **0.02** | 134 (27) |
| **Clopidogrel/Ticagrelor (Y)** | 12 (8) | 16 (7) | 10 (9) | 0.8 | 39 (8) |
| **Anticoagulation (Y)** | 27 (17) | 43 (19) | 17 (16) | 0.72 | 87 (18) |
